# Retraction: Myeloid ecotropic viral integration site 1 inhibits cell proliferation, invasion or migration in human gastric cancer

**DOI:** 10.18632/oncotarget.28649

**Published:** 2024-09-17

**Authors:** Fei Song, Hong Wang, Yingying Wang

**Affiliations:** ^1^Department of General Surgery, Shandong Provincial Third Hospital, Jinan, Shandong, China; ^2^Department of Gynecologic Oncology, Shandong Cancer Hospital Affiliated to Shandong University, Shandong Academy of Medical Sciences, Jinan, Shandong, China

**This article has been retracted:** In Figure 1A and 1D, two MEIS1 bands were used to describe a GAPDH control. In Figure 6B, the same band is presented as BAX and Survivin at the same time. In addition, in Figure 2, panels 2C and 2D, all the micrographs duplicate those in an earlier paper describing completely different conditions [1]. Therefore, the Editorial decision was made to retract this paper. All authors agreed with the decision.

Original article: Oncotarget. 2017; 8:90050–90060. 90050-90060. https://doi.org/10.18632/oncotarget.21376
